# Software Tools and Approaches for Compound Identification of LC-MS/MS Data in Metabolomics

**DOI:** 10.3390/metabo8020031

**Published:** 2018-05-10

**Authors:** Ivana Blaženović, Tobias Kind, Jian Ji, Oliver Fiehn

**Affiliations:** 1 NIH West Coast Metabolomics Center, UC Davis Genome Center, University of California, Davis, CA 95616, USA; iblazenovic@ucdavis.edu (I.B.); tkind@ucdavis.edu (T.K.); 2 State Key Laboratory of Food Science and Technology, School of Food Science of Jiangnan University, School of Food Science Synergetic Innovation Center of Food Safety and Nutrition, Wuxi 214122, China; jijianjndx@126.com; 3 Department of Biochemistry, Faculty of Sciences, King Abdulaziz University, Jeddah 21589, Saudi Arabia

**Keywords:** tandem mass spectrometry, library search, in silico fragmentation, high resolution mass spectrometry, compound identification, metabolomics

## Abstract

The annotation of small molecules remains a major challenge in untargeted mass spectrometry-based metabolomics. We here critically discuss structured elucidation approaches and software that are designed to help during the annotation of unknown compounds. Only by elucidating unknown metabolites first is it possible to biologically interpret complex systems, to map compounds to pathways and to create reliable predictive metabolic models for translational and clinical research. These strategies include the construction and quality of tandem mass spectral databases such as the coalition of MassBank repositories and investigations of MS/MS matching confidence. We present in silico fragmentation tools such as MS-FINDER, CFM-ID, MetFrag, ChemDistiller and CSI:FingerID that can annotate compounds from existing structure databases and that have been used in the CASMI (critical assessment of small molecule identification) contests. Furthermore, the use of retention time models from liquid chromatography and the utility of collision cross-section modelling from ion mobility experiments are covered. Workflows and published examples of successfully annotated unknown compounds are included.

## 1. Introduction

Metabolomics is the comprehensive study of small molecules present in cells, tissues and body fluids. Advances in metabolic profiling have led to discoveries of biomarkers in a variety of medical conditions using metabolomics and lipidomics approaches, including the vision to utilize metabolomics for precision medicine [1,2,3]. Untargeted metabolomics experiments allow for the acquisition of thousands of metabolite signals in a single sample [4]. However, a large percentage of these signals remain structurally unknown [5], and therefore compound identification remains one of the large obstacles in metabolomics [6,7].

Currently, two major analytical platforms are used in the small molecule identification process. Nuclear magnetic resonance (NMR) is a powerful structure elucidation technique and it has a significant advantage due to its nondestructive and noninvasive characteristics of analysis. However, this method lacks the sensitivity needed for the simultaneous analysis of thousands of metabolites observed in biological samples [8,9]. High resolution chromatographic separation techniques coupled to accurate tandem mass spectrometry (LC-MS/MS) represents the most important metabolomics platform. This technology allows for the physical separation of thousands of metabolites and therefore provides a more comprehensive view of the metabolome.

Classical structure elucidation using NMR commonly elucidates the full structure using de-novo approaches [10]. The natural product [11], environmental [12] and mass spectrometry community [13] usually have different definitions for compound identification. In metabolomics, five different levels exist (see Table 1) including the new ‘Level 0’ that requires the full 3D structure and stereochemistry information. More common are ‘Level 1’ annotations that are confirmed by two orthogonal parameters, such as retention time and MS/MS spectrum. These levels were initially forged by the Metabolomics Standards Initiative (MSI) of the Metabolomics Society [14,15] and were later refined by the compound identification workgroup of the society. It is recommended to integrate the level of annotation for each compound into metabolomic profiling reports.

**Figure 1:**
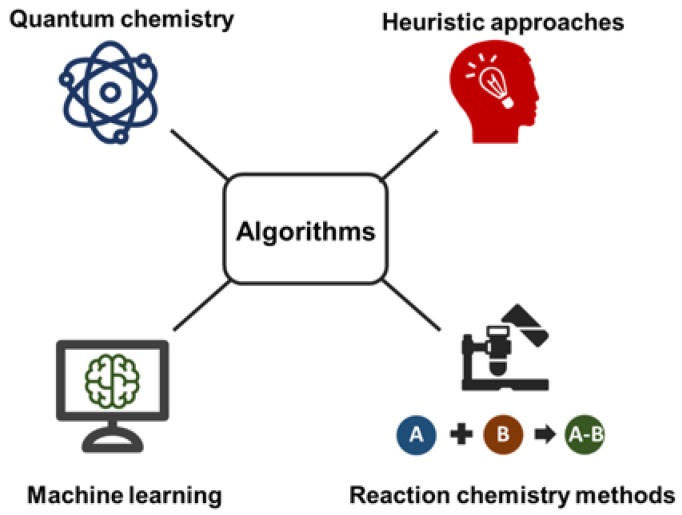
Computational metabolomics approaches help to unravel the complexity of the metabolome and especially shed light on unknown metabolites. This includes technologies across different disciplines, including quantum chemistry, machine learning, heuristic approaches and reaction chemistry-based methods.

A number of reviews have been published that cover many diverse metabolomics topics including chromatography, data processing and statistics in great detail [16,17,18,19,20,21,22,23,24]. We mostly focus on papers that discuss structure elucidation approaches involving liquid chromatography tandem mass spectrometry (LC-MS/MS) within the last 5–10 years. The review is thematically divided into important sections that include mass spectral database search, in silico fragmentation tools and orthogonal coupled techniques including retention time matching and ion mobility spectrometry (see Figure 1). Lipidomics and mass spectral imaging approaches are not fully covered. Classical chemical derivatization and isotope labeling studies are discussed elsewhere [25]. Here, we only discuss a selected number of software tools and databases than can help practitioners to obtain results during the annotation of unknown compounds; larger surveys were covered in [17,23,26].

## 2. Compound Databases and Chemical Space

The chemical space of small molecules currently covered in databases such as PubChem, ChemSpider or the Chemical Abstracts Database is larger than 120 million compounds [16] (see Table 2). The number of compounds with biological relevance is estimated at 1–2 million [27]. However, a large majority of metabolites discovered during untargeted metabolic profiling remains unknown, including many microbial [28], environmental [29] and natural compounds. In fact, very few reports in published research have more than 20% identified compounds in untargeted analysis, as can be seen at the Metabolomics Workbench [30], or the European metabolomics repository MetaboLights [31].

During the structure elucidation process, small molecule databases serve as a foundation of known and well-researched metabolites (see Table 2). Enzyme and pathway databases such as KEGG, MetaCyc and BRENDA serve as connectors to the proteomics and transcriptomics domain. Molecular formulas or accurate masses can be queried in such databases, and potential structure candidates can be retrieved to be investigated by in-silico fragmentation software tools. In many cases, it is important to restrict the search space by including taxonomy information. Molecular discovery in humans can be obtained from the Human Metabolome Database (HMDB) [37], and plant researchers should restrict their search space to primary and secondary plant metabolites such as found in the UNPD (Universal Natural Product Database) database [39] or compounds covered in the natural product space [41,42]. For exposome related research, environmental database resources can be utilized [43,44].

In case the compounds have not yet been described in the literature, enzymatic expansion databases such as MINES (Metabolic in silico Network Expansion Databases) can be searched (http://minedatabase.mcs.anl.gov/). MINES covers over 500,000 substances derived from KEGG and other pathway databases by applying known enzymatic transformation rules [40]. These novel compounds are not covered in traditional databases such as PubChem but can be utilized as hypothesized starting molecules for structure elucidation [45].

## 3. Mass Spectral Database Search for Fast Annotations

Mass spectral database search is currently the fastest and most accurate way for initial compound annotations. Current public and commercial mass spectral databases contain around 1–2 million spectra of one million unique compounds. Most of these spectra are EI mass spectra for GC-MS, while fewer are available for LC-MS/MS analysis. Traditionally, these databases have been derived from authentic experimental reference compounds and were collected from the literature [46]. Lately, computationally generated in silico spectra have also gained in importance, as discussed below. The experimentally derived as well as the in silico generated databases are enriched with metadata such as instrument types, collision energies, ionization mode and structural information such as the InChIKey [47] and SPLASH (spectral hash code) for uniqueness calculations [48]. Both InChIKey and SPLASH are important as unique identifiers in the structural and spectral domain. Errors during reference library building can be curated using software or manual data correction [49]. Table 3 lists a selection of commonly used mass spectral databases, see recent reviews for a complete coverage of mass spectral databases [19,50].

In terms of coverage, up to 400 metabolites were identified from NIST plasma reference standards utilizing multiple platforms and database matching [56]. The NIH Common Fund metabolomics ring trial with the participation of multiple US labs annotated around 1000 metabolites using multiple technologies and reference spectra matching. However, literature references for the plasma or serum metabolome covered up to 5000 compounds by combining targeted and non-targeted metabolomics analysis from five platforms [57]. It is therefore clear that matching experimental reference spectra to experimental reference databases is a severely limited process and covers only a fraction of the detectable metabolome.

Many modern algorithms for peak detection and mass spectral deconvolution have in-built database search algorithms. That includes freely available search algorithms such as the NIST MS Search GUI (graphical user interface), NIST MS PepSearch or MS-DIAL [58]. Commercial software from mass spectrometry vendors use similar algorithms.

Scoring mass spectra has been traditionally performed by a number of algorithms such as probability match searching, dot-product search and other similarity measures [19]. Recently, a novel hybrid similarity search method has been introduced that can annotate unknown spectra. The method does not account for the precursor *m*/*z* and instead utilizes similar neutral losses and fragmentation patterns [59]. Spectral similarity can also indicate structural similarity and this information can be used for annotation of unknown compounds [60]. Clustering approaches that use the cosine similarity of product ion spectra by clustering structurally similar compounds can improve the annotation of unknown metabolites [61]. Despite the advantages of a fast library search, it is becoming clear that mass spectral scoring algorithms have to be improved, especially for product ion spectra that contain only few fragments [46] or for those libraries that integrate spectra from multiple instrumentation types. Here, approaches that can calculate false discovery rates (FDR) will be useful to improve spectral match and annotation quality [62,63].

Community efforts have positively impacted the sharing of mass spectra. The MassBank database (http://massbank.jp) is one of the most successful examples, with a wide user base and contributors from many different countries [52]. In a coalition of database servers, the European MassBank efforts (https://massbank.eu/) [64] and MassBank of North America (http://massbank.us/) enable immediate sharing of mass spectra of annotated structures, including autocuration of spectra and chemical structure information (InChI keys). In comparison, the GNPS [54] spectral database utilizes crowd sourcing approaches to annotate unknown compounds. Commercial libraries such as NIST17 still play an important role because of high levels of manual curation, overall good data quality and wide coverage of substances.

## 4. In Silico Generation of Mass Spectra and MS/MS Spectra

As described before, scientists today have access to around 100 million known compounds in PubChem and ChemSpider. However, fewer than one million compounds have associated electron ionization (EI) mass spectra (for GC-MS applications), and even fewer LC-MS/MS tandem mass spectra are available. Generating in silico mass spectra, therefore, is a unique opportunity to close this gap. Research into computational generation of mass spectra has gained much traction during the last five years. Four general methods can be distinguished: quantum chemistry, machine learning, heuristic-based methods and chemical reaction-based methods.

Quantum chemistry methods use first-principles and purely physical and chemical information to generate mass spectra. In a major breakthrough for computational mass spectrometry, Grimme described in 2013 how Born–Oppenheimer ab initio molecular dynamics can be used to generate in silico electron ionization mass spectra of any given compound [65,66,67,68,69]. An overview of methods for in silico generation of mass spectra, including commercially or freely available algorithms is listed in Table 4.

Machine learning-based methods such as CFM-ID developed by Allen et al. allow for the computation of CID-MS/MS [70] and EI-MS spectra [71] directly from molecular structures. It is a very versatile approach useful for small molecules and peptides up to 1000 Da [72]. The methodology requires diverse and large training sets which subsequently will improve overall accuracy during training.

Heuristic approaches such as LipidBlast are advantageous for compound classes that have reoccurring and predictive fragmentations such as lipids [73]. However, the heuristic approach cannot be expanded to molecules with very diverse structural scaffolds. The libraries themselves can be easily extended to include novel or recently discovered lipid classes [40,45,74].

Reaction-based approaches are covered in the Mass Frontier software (HighChem Ltd., Bratislava, Slovakia) and based on thousands of reactions discovered in the literature. Novel molecules can be fragmented based on observed reaction pathways. Only bar code spectra can be generated, hence peak abundances are missing.

The accuracy of in silico generated peaks and their abundances have to be largely improved. A comparison between QCEIMS and CFM-ID has shown that both algorithms perform well enough to get correct identifications for half of the 61 investigated molecules [75]. However, certain rearrangement reactions, including McLafferty rearrangements, remain underestimated. The highly accurate and fast OM2 and OM3 semiempirical methods [76] have been further improved by the GFN-xTB Hamiltonian into QCEIMS [77]. Independent approaches described DFT reaction pathway and transition state modelling to model EI mass spectra [78] or Monte Carlo sampling to obtain EI mass spectra for select cases [79].

Currently, there is no fully automatic software for the generation of in silico MS/MS spectra based on LC-MS collision induced dissociation (CID). Several groups have shown interest in this challenging topic and have provided steps that can finally lead to a fully automated stand-alone solution. That includes workflows to automatically find the correct protonation sites in a molecule [80,81], ways to utilize rotamers, conformers, Boltzmann averaging and the evaluation of semiempirical and density functional methods (DFT) to calculate fragments.

The validation of generated in silico spectra is probably the most crucial aspect, especially when ‘blindly’ applying software models to large molecule repositories. For example, the original CFM-ID models were trained on a number of small metabolites. Therefore, these initial models are focused on lower molecular weight molecules and may not be feasible for the generation of in silico spectra of high molecular weight lipids or large complex secondary metabolites. In order to obtain high accuracy, the CFM-ID models have to be retrained with adequate lipid and secondary metabolite training sets. As always, external validation with mass spectra that were not available during training is highly recommended. For ab initio models, large validation sets with thousands of compounds have to be generated to obtain confidence scores.

Furthermore, regarding in silico spectra, two major problems will arise in the future. First, calculational processes follow the normal distribution; hence a large number of average accuracy in silico spectra will be observed. The flanks will consist of a small number of inaccurate spectra as well as a small number of high-quality spectra. Here, research needs to focus on ways to improve the average accuracy of in silico spectra predictions, but also to exclude such low-quality in silico spectra. In addition, the community will need to develop improved MS/MS match confidence scores. Otherwise, wrong spectra and publications with false compound annotations lead to many false-positive annotations in databases. The second problem is the generation of millions of very similar in silico spectra, because compound databases host millions of structurally very similar compounds. This will lead to an effect called database poisoning, filling mass spectral databases with compound spectra that cannot be easily distinguished by database search alone. Here, research has to focus on orthogonal filtering methods such as ion mobility or retention time filters.

## 5. In Silico Fragmentation Software

**Figure 2:**
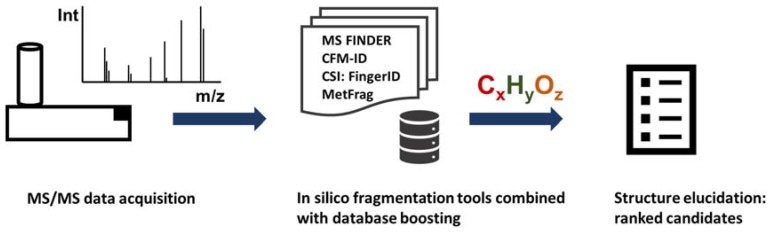
In silico fragmentation tools such as MS-Finder, CFM-ID, CSI:FingerID and Metfrag utilized known compounds from structure databases to calculate fragments compare those theoretical fragmentations against experimental spectra. When combined with MS/MS database search and utilizing additional metadata annotation rates can be increased tremendously.

In silico fragmentation approaches for the annotation of unknown molecules are used in those cases where no reference mass spectra are available for database matching [82]. These generally involve matching experimental spectra against a selection of in silico generated fragments calculated on candidates retrieved from known compound databases (see Figure 2). Instead of searching mass spectral databases which cover only one million compounds, in silico fragmentation algorithms have access to molecular structure databases including ChemSpider and PubChem covering almost 100 million compounds [83].

These in silico fragmentation approaches aim to identify “known unknowns”—i.e., compounds present in molecular structure databases but without any reference spectra—by calculating a score between the experimental spectra and the predicted spectra (or predicted fragments). The major disadvantage is that “unknown–unknown” compounds cannot be elucidated in such a way. Below, we discuss some of the tools that have participated in structure elucidation challenges and can be used for batch annotations of unknown compounds (see Table 5). Additional software including iMet [84], MAGMa [85], MIDAS [86] and Midas-G [87] are discussed elsewhere. Most of the approaches below have been discussed in much greater technical detail in a series of excellent reviews [88,89,90,91].

MetFrag [92] is a combinatorial fragmenter that retrieves candidate structures from PubChem, ChemSpider, KEGG, and a few other more specific compound databases. Candidates are fragmented using a bond dissociation approach and are finally matched to experimentally obtained spectra. MetFrag and MetFusion [93] have been actively developed and improved, allowing local or web-based use. The LipidFrag tool was developed later to increase confidence in lipid annotations [94].

MS-FINDER [84] is a Windows based GUI software aiding the structure elucidation process by in silico fragmentation of all predicted molecular formulas, determined from the accurate mass, isotope ratio, and product ion information [95], which are retrieved from 15 databases that are embedded into MS-FINDER [96,97]. The structures are then ranked by variety of factors including nine hydrogen rearrangement rules as the most contributing factor to the final score calculations.

CSI:FingerID [98] is a freely available web-service and uses a two-step scheme: first, a kernel-based approach is utilized to predict molecular fingerprints [99] from its MS/MS spectrum and then the predicted molecular fingerprints are matched against a molecular compound database. Included is a module that combines computation and comparison of fragmentation trees for the prediction of molecular properties of the unknowns as well as the molecular formula generation. Novel algorithms such as IOKR (input output kernel regression) [100] are now integrated into the workflow. The stand-alone SIRIUS GUI software [101] is used to calculate fragmentation trees and, subsequently, molecular formulas [102]. SIRIUS is now directly coupled to the CSI:FingerID online server that matches fingerprints against a database and retrieves ranked structure candidates.

CFM-ID (competitive fragmentation modeling) is a suite of software tools that can perform spectra prediction and compound identification. It is based on a machine-learning approach including chemical rules andva is available for ESI MS/MS data as well as EI mass spectra. CFM-ID can be used as a web server or can be called locally through command line utilities on Windows, Linux and MacOS. For larger datasets, the software can be deployed to clusters to reduce the computational times.

ChemDistiller [103] is a Python-based tool that uses structural fingerprints and fragmentation patterns together with a machine learning algorithm to annotate unknown compounds. It utilizes multiple target databases covering more than 130 million compounds to annotate unknowns and the output is presented in a web interface for further inspection. It is a very fast and highly parallelized tool that makes use of modern multi-core CPUs.

Mass Frontier [82], developed by HighChem, is based on observed experimental gas-phase fragmentation reactions. It contains basic fragmentation rules as well as an exhaustive library of over 100,000 known fragmentation rules collected from published data which also allows for fragmentation predictions and annotation of unknowns [104]. The software supports electron ionization (EI) and collision induced dissociation (CID) ESI MS/MS modes. Mass Frontier can search internal databases or the mzCloud database and is commercially available.

To improve the annotation rates, database type restrictions such as environmental, plant, metabolic pathway databases can be applied. Taxonomy restrictions are also useful when researching specific organisms. Generally, in silico fragmentation algorithms still need to improve tremendously. A comparison of four algorithms using the CASMI test compounds as input has shown that pure in silico algorithms could only identify 17–25% of the compounds correctly [105]. Boosting the output by adding MS/MS search and bio-database focused lookups as well as combining the outputs of multiple software tools led to much higher identification rates of up to 93% accuracy [106]. Combining multiple in silico fragmentation software with a-priori information is a valuable option when facing a structure elucidation challenge [106].

## 6. Retention Time Prediction

Retention times are important as orthogonal filters during the structural determination in metabolic profiling experiments. A number of MS/MS and retention time databases have been developed for metabolic profiling [55]. However, these tools usually contain only a few hundred experimentally obtained retention time values. It is therefore useful to predict theoretical retention times utilizing the millions of existing compounds in compound databases by quantitative structure-retention relationship (QSRR) modelling [107]. This field of research has been active for more than 30 years. Traditionally, group-contribution methods were used for GC-MS modelling by assigning small retention index increments to specific substructures [108]. However, a vast amount of different separation columns and an infinite combination of solvent buffer systems and chromatographic conditions exist in LC-MS, locking the predicted models to very specific conditions [109].

Another major reason why there is no universal retention prediction method for LC-MS/MS is the lack of large and diverse training sets. A minimum of a thousand compounds covering all major chemical scaffolds in hydrophilic interaction liquid chromatography (HILIC) or reversed-phase chromatography (RP) are required to generate a robust retention prediction model useful for metabolic profiling.

An additional important consideration for retention time models is the applicability domain or structural space used in model building [110]. In short, if a natural product training set is used, it should be used for the prediction of natural product predictions and not for drugs. A simple measure would be to perform a principal component analysis on the substructure feature space for training samples and new predictive compounds and to confirm that the space overlaps. However, a recent approach utilized 1955 synthetic screening compounds that cover a similar scaffold space as small metabolites and used artificial neural networks to predict LC-MS retention indices for 202 endogenous metabolites [111]. This approach is particularly interesting because plated screening compounds are commonly less expensive than endogenous metabolites. By massively increasing the structural scaffold space, the retention model can become more robust, even if these molecules will never be annotated in biological samples. Many retention time prediction models are usually locked to a specific LC column and a solvent and buffer system, unless a “retention projection” method can be applied to transfer data to other chromatographic systems [112,113,114].

Retention times can be predicted by using chemical descriptors as input parameters which can be computed directly from structures by tools such as Dragon [115], MOLD2 [116] or PaDel [117]. Dragon 7 now calculates 5270 molecular descriptors, covering fragment counts, topological and geometrical descriptors. Low-energy three dimensional conformer structures can be generated by a number of tools [118] and even better with quantum chemical methods [119]. Subsequently, regression models can be built using the descriptor data as input and the retention time as a target function. Over 200 machine learning models, preferably with deep neural networks [120] or fast random forest methods [121], are now available. To improve accuracy and prediction power, complex gradient boosting methods (XGBoost/LightGBM) and ensemble methods such as bagging, stacking and averaging are now routinely employed [122]. In the past, a wide variety of retention prediction models have been proposed for HILIC and reversed phase columns based on different machine learning approaches. These included partial least square methods [123,124,125], multiple linear regression [126,127,128], support vector regression [129,130], random forests [131] and artificial neural networks [132,133,134].

In summary, the success of the retention time modelling depends on the size and the diversity of the compound training data set. Currently, most RT models are locked to specific columns and conditions, unless a retention projection method is used. For useful retention time prediction models, the only remedies are large and diverse training sets covering multiple compound classes to obtain reliable, highly predictive and accurate models.

## 7. Ion Mobility and the Use of Collision Cross Section (CCS) Values

LC-MS/MS alone will often be unable to discriminate between stereoisomers and regioisomers, unless chiral columns are utilized. It is therefore useful to couple ion mobility analyzers to LC-MS/MS to allow for a higher number of features to be separated and detected [135]. Ion mobility is a technique that separates ions in an inert buffer gas (nitrogen, hydrogen) under the influence of an electric field [136,137]. Several types of ion mobility analyzers are available, among them drift tube ion mobility (DTIMS), traveling wave ion mobility spectrometry (TWIMS) and FAIMS [138].

For DTIMS and TWIMS, the observed drift times are influenced by relative molecule size and conformational parameters. For DTIMS, cross-section values (CCS) can be directly measured and computed [139,140], and for TWIMS the CSS values can be obtained from calibrations with known standards [141]. The FAIMS technology has limited peak capacity [142,143], but can be used as an orthogonal filter to separate different classes of compounds and to improve signal/noise ratios during measurements [144]. For FAIMS, no collision cross-section values (CCS) can be determined [138].

**Figure 3:**
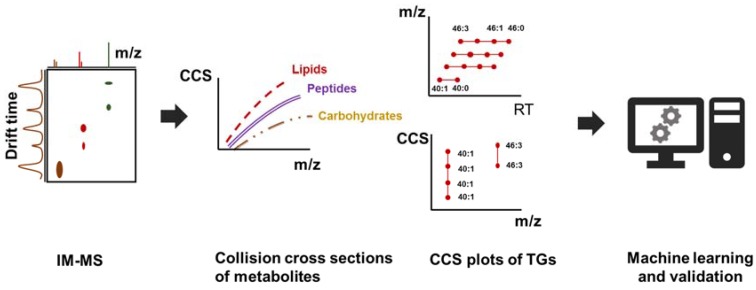
Ion mobility can be used as an additional orthogonal approach to resolve complex mixtures. The experimental collision cross-section values (CCS) can be further utilized to train machine learning models to further enrich compound databases with CCS information.

The experimental CCS values have a very high reproducibility and CCS values with relative standard deviation (RSD) of <1–2% can be routinely obtained [139,145,146]. This opens up the LC-IMS-MS/MS technology for orthogonal filtering approaches utilizing CSS values [147] (see Figure 3) and more importantly for predictive technologies utilizing CCS values in a similar to retention time predictions. Such predictive approaches can include computational and quantum chemical models [148,149] as well as machine learning predictions [150] such as artificial neural networks [132,151]. Prediction errors as low as 3% have been reported for CCS models [152]. Once these models are applied to structures from large metabolomic databases, they can be used for filtering during the compound identification process [138,153], and such predicted values are covered in publicly available databases such as MetCCS [152] or LipidCCS [154,155]. Currently, an estimated total of 3000–4000 experimental small molecule CCS values have been reported in a recent review [150] with the largest single collection containing CCS values for 1420 compounds [145]. Focused collections for sterols [156], metabolites and xenobiotics are also available [139,157].

Several considerations have to be taken into account when working with CCS values and predictive databases. CCS values of individual compounds depend on many additional parameters such as buffer gas, solvents, temperature, pH, ion activation voltage and conformer/rotamer ensembles [158,159]. For example, different ion species such as [M + H]^+^ and [M + Na]^+^ have different CCS values, differing on average ±7 Å^2^ based on values obtained from [139]. This is related to conformational changes and subsequently leads to the conclusion that different adducts have to be modelled and predicted separately. Furthermore, different protonation sites or protomers can lead to different CCS values [145]. Drugs such as benzocaine can have N- or O-protonated forms leading to different CCS values for the same compound [160]. The different protomers can be determined with the help of quantum chemical methods [161,162] and cheminformatics methods that calculate different protonation sites. Reference standards themselves may not be enantiomerically pure and therefore can lead to measurement of multiple experimental CCS values. Furthermore, while CCS values predicted on the same instrument type have low RSD measurement errors <1% [163], the experimental CCS values may differ between different instrumental setups (DTIMS/TWIMS), as well as prediction models. The drug Indomethacin for the proton adduct has a reported CCS value of 183.54 Å^2^ measured on a drift tube IMS (DTIMS) [139]; the same compound has a CCS value of 179.039 Å^2^ measured on a TWIMS setup, and the predicted value is 197.7 Å^2^ and therefore falls outside the 3% median prediction error [164].

Because of the IMS capability of separating stereoisomers and other isobaric compounds, the routine use of CCS values will become more and more important. The excellent experimental reproducibilities of CCS measurements compared to retention times will also improve identification rates. Once larger CCS datasets become publicly available, they can be combined, average consensus values can be calculated and CCS prediction methods can be retrained with larger compound numbers and therefore will automatically become more accurate. Technological advances such as printed circuit board (PCB)-based devices led to ion elevators and escalators in multilevel structures [165]. Therefore, such structures for lossless ion manipulations (SLIM) have demonstrated unprecedented ultra-high resolution ion mobility [166].

## 8. Compound Identification: Hybrid and Orthogonal Approaches

The following section discusses some general compound identification workflows as well as a few selected cases of single compound identification examples via mass spectrometry. Workflows are important for highly reproducible and repeatable metabolomics analysis. Among those are Galaxy workflows [161] such as Workflow4metabolomics.org, as well as Taverna and KNIME workflows, but with a considerably lower user base [167]. A conceptual compound ID workflow has been described that includes in silico metabolic synthesis, in silico fragmentation [168] and finally annotation of compounds via database scoring [169]. The same paper discusses the importance of meta-integration of multiple tools and multiple layers of information to improve confidence in compound identification. Another related review discusses the importance of inclusion of MS^1^ peak relationships such as adducts and neutral losses, the inclusion of MS/MS data and biochemical knowledge as well as modelling of retention times as an orthogonal filter. A knowledge-based workflow for metabolite annotations that includes ionization rules, adduct formation rules and retention time rules was described in [170].

However, even in-source fragmentation LC-MS mass spectra when used together with retention times of authentic compounds can be sufficient for ‘Level 1’ annotations in metabolomics [171]. A pipeline that uses multicriteria scoring, including retention times, intensity profiles and adduct patterns was developed for high-resolution mass spectral data [172]. The extraction of common occurring substructures from MS/MS data can help during higher level annotations [173]. Another workflow included the use of multiple identification criteria such as accurate mass, retention time, MS/MS spectrum, and product/precursor ion intensity ratios to support reversed phase and HILIC based metabolic profiling [174]. Two in silico fragmenters and two retention prediction models were utilized to annotate hydrophobic compounds [175]. A tool for improved and automated adduct detection was discussed in [176], leading to 83% correct annotations of adduct ions. The dereplication of natural products with the help of a fragment database was described in [177]. Pitfalls, limitations and general recommendation during data processing and compound identifications were discussed in [24,178,179].

Full structure elucidation of single novel compounds with chromatography and mass spectrometric analysis is possible but is harder than with the isolation of compounds and NMR analysis. A clear benefit of LC-MS/MS approaches is the limited amount of material needed, in comparison to LC-MS/MS-NMR methods. A recent report annotated N^1^-acetylisoputreanine and N^1^-acetylisoputreanine-gamma-lactam by metabolic profiling and used custom synthesis to confirm the commercially unavailable metabolite [180]. Another approach used multiple-stage tandem mass spectrometry (MS^4^) and custom synthesis to identify and confirm *N*,*N*,*N*-trimethyl-l-alanyl-l-proline betaine in human plasma. Novel glycolipids were found in yeast annotated by combining multiple mass spectrometric platforms and chiral chromatography to ascertain stereoisomer configuration [181]. Another approach showed the combined use of high-resolution MS/MS data and use of the metabolic in-silico network expansion database (MINE) for the discovery of novel methylated epi-metabolites including N-methyl-UMP [45]. Natural products can be manually annotated with high success rates [182], but such approaches require deep mass spectral knowledge. In the future, such manual approaches must be translated into practical expert-algorithms and software that allows non-experts to perform such complicated analysis to a certain degree [27]. Finally, all pipelines and workflows must be validated by independent and external benchmark sets such as the CASMI competitions discussed below.

## 9. Critical Assessment of Small Molecule Analysis (CASMI)

The CASMI (critical assessment of small molecule identification) contest (http://www.casmi-contest.org) has been held since 2012 as a worldwide scientific competition to determine the best approaches for identifying small molecule structures directly from mass spectra [183,184]. The competitions are commonly structured into different categories, including best natural product determination [96,182,185], best molecular formula determination [186] and unknown compound determination. More recently, categories that allow for in silico fragmentation software only [187] and a category that allows for all meta-data use were included [85]. Participants publish their findings in special journal issues selected by the CASMI organizers and describe how they implemented and performed their structure annotation processes.

The latest CASMI 2017 contest featured 300 small molecule challenges and may continue to serve as a test bed for the performance and comparison of software tools and pipelines. On the other hand, many research papers describe approaches and pipelines that focus on a few selected “cherry picked” test cases. Therefore, it is recommended for groups that develop compound identification software to participate in the yearly CASMI contests to showcase the performance of their software against others. Best of all, any published article about novel approaches or software tools should participate in the CASMI small molecule identification contests or at least use former CASMI data sets for validation of the approaches used.

Future CASMI contests may be held in a completely automatic fashion, as long as the software and pipeline are fully publicly available. One idea would be to make these tools so easy to use that non-specialists from the broader community can utilize them quickly and improve compound identification rates. The increasing number of challenges and CASMI participants shows that the field of unknown-identification is moving steadily forward.

## 10. Data Sharing and Data Retention

Sharing research data and software helps to validate the claims made in publications and, more importantly, lets researchers freely reuse that data and develop novel research ideas [188]. Unfortunately, while journals support data sharing, they often do not strictly enforce it [189]. Here, funding agencies such as the National Institutes of Health (NIH) in the United States have a large leverage to make data sharing mandatory. Both NIH and the US National Science Foundation (NSF) require data retention and data sharing plans for grant proposals, cultivating a way for better reuse of research data. Currently, funding organizations worldwide do not strictly enforce the public sharing of metabolomics data. This is contrary to genomics, where deposition of genomic data is required before any publication.

For computational tools and software, it is recommended to use public software repositories such as GitHub, BitBucket and SourceForge services (see Table 6). In this case, repositories can be forked (copied) and multiple copies remain even when the original distributer does not support them anymore.

For metabolomics data sets, the Metabolomics Workbench [30] or the European metabolomics repository MetaboLights [31] should be considered. These repositories contain a high level of metadata information, which requires a high level of data preparation before the upload process. The advantage is that experiments are very well described and that such metadata can be queried at a later time point. The incentive of the GNPS repository [54] is that mass spectra of many unknown compounds are collected, and identification of such spectra might be enhanced through community efforts. The OpenMSI [190] and Metaspace.eu [63] projects provide open analysis solutions for mass spectral imaging data. Scientific data sets from all branches of research can be submitted to the Zenodo research repository, which also supports citable digital object identifiers (DOI). The long-standing effort of collecting freely available mass spectra of pure reference compounds at MassBank (Japan) has now been complemented by collaborative efforts in the USA (MassBank of North America, MoNA) and the Norman MassBank in Europe. Due to the allowed unrestricted use, open spectral collections can be used for algorithm training in open or commercial software.

Specifically, the MoNA database has an advantage of automated spectral uploads via REST API, which allows for instantaneous sharing of novel compounds and associated spectra. MoNA collates all worldwide publicly available mass spectra, including spectra from MetaboBASE, GNPS, HMDB, LipidBlast, ReSpect and MassBank spectra in one unique repository. Users can freely download spectra based on metadata tags, including based on instrument, vendor, mass accuracy, types of chromatography, or based on compound classes (supported by ClassyFire) [191].

The publication of tools or databases that are neither publicly nor commercially available should be avoided. Such opaque software does not contribute much to the field and cannot be validated independently. We therefore mostly refrained from referencing such publications or tools in this review. Software tools should be validated on public, large and diverse datasets before making claims that they outperform any other tool.

## 11. Conclusions and Outlook

Computational metabolomics strategies for compound identification have gained increased attention in the community. Unknown metabolite signals cannot easily be used for biological interpretations [7], and increased efforts and validations for compound identifications are critical for the field to move forward. Approaches that do not require the identification of metabolic features should be used with extreme caution because they may lead to false interpretations. The identification of metabolites with a high level of confidence is required in order to improve metabolomics applications in the field of translational and clinical research.

Bioinformatics researchers have helped the proteomics and genomics community over many years to solve problems in their domain. However, the bioinformatics community had a smaller impact on the small-molecule community due to the chemical structure-centric approaches that are needed for structure elucidation in metabolomics. To this end, the much smaller cheminformatics community still struggles to provide adequate support simply due to its much smaller size and impact. Therefore, collaboration with researchers from scientific branches such as machine learning and the quantum chemistry community need to be actively embraced. The computational metabolomics community is a quite small but innovative community, and many more research groups worldwide contribute now in friendly competition.

## Abbreviations and Glossary

MS^n^Multiple stage mass spectrometryCASMICritical Assessment of Small Molecule IdentificationCCSCollisional cross-sectionCFM-IDCompetitive Fragmentation Modeling for Metabolite IdentificationFAHFAsFatty Acid ester of Hydroxyl Fatty AcidsFragmentation treeMass spectral fragmentation pathway of a compoundGNPSGlobal Natural Products Social molecular networkingHMDBHuman Metabolome DatabaseIMIon mobilityInChIKeyHash key or short unique structure codeLipidBlastIn silico generated database for lipid identificationMassBankMass spectral databaseMetaboBASEMass spectral library developed by BrukerMoNAMassBank of North AmericaNISTNational Institute of Standards and TechnologyNMRNuclear Magnetic ResonanceReSpectRIKEN MSn spectral database for phytochemicalsSPLASHHashed code or unique identifier for mass spectra

## Figures and Tables

**Table 1:** New confidence levels of compound annotations, as discussed by the Compound Identification work group of the Metabolomics Society at the 2017 annual meeting of the Metabolomics Society (Brisbane, Australia). The new addition refers to the ‘Level 0’ annotation; other levels remain as discussed by the Metabolomics Standards Initiative.

Confidence Level	Description	Minimum Data Requirements
Level 0	Unambigous 3D structure: Isolated, pure compound, including full stereochemistry	Following natural product guidelines, determination of 3D structure
Level 1	Confident 2D structure: uses reference standard match or full 2D structure elucidation	At least two orthogonal techniques defining 2D structure confidently, such as MS/MS and RT or CCS
Level 2	Probable structure: matched to literature data or databases by diagnostic evidence	At least two orthogonal pieces of information, including evidence that excludes all other candidates
Level 3	Possible structure or class: Most likely structure, isomers possible, substance class or substructure match	One or several candidates possible, requires at least one piece of information supporting the proposed candidate
Level 4	Unkown feature of insterest:	Presence in sample

**Table 2:** Overview of selected compound databases commonly used for compound identification.

Database	Targets	Description
PubChem [32]	All small molecules	Small molecules, metadata
ChemSpider [33]	All small molecules	Small molecules, curated data
KEGG [34]	Metabolites	Pathway database, multiple species
MetaCyc [35]	Metabolites	Pathway database, multiple species
BRENDA [36]	Enzymes	Enzyme and metabolism data
HMDB [37]	Metabolites	Human metabolites
CHEBI [38]	Small molecules	Molecules of biological interest
UNPD [39]	Metabolites	Secondary plant metabolites
MINE [40]	Metabolites	In silico predicted metabolites

**Table 3:** Overview of selected mass spectral databases commonly used for compound annotations. Specialized reviews that cover other mass spectral databases are referenced in the text.

Database	Targets	Description
NIST	EI-MS, CID-MS/MS	Curated DB, graphical interface
WILEY	EI-MS, CID-MS/MS	Largest collection of EI-MS data
METLIN [51]	CID-MS/MS	Developed for QTOF instruments
MoNA	EI, MS/MS, MSn	Autocurated collection of spectra
MassBank [52]	EI, MS/MS, MSn	Longest standing community database
mzCloud [53]	MSn	Multiple stage MSn
GNPS [54]	MS/MS	Community database
ReSpect [55]	MS/MS, RT	Plant metabolomics database

**Table 4:** Overview of methods for in silico generation of mass spectra, including commercially or freely available algorithms. Additional tools are referenced in text.

In Silico Method	Software	Platform	Description
Quantum chemistry	QCEIMS	EI-MS	Uses chemistry first principles; requires cluster computations
Machine learning	CFM-ID/CSI:FingerID	EI-MS CID-MS/MS	Requires diverse training sets; Fast method
Heuristic approaches	LipidBlast	CID-MS/MS	for specific compound classes (lipids); Fast method
Reaction chemistry methods	MassFrontier	EI-MSCID-MS/MS	generates only bar code spectra; Covers experimental gas phase reactions

**Table 5:** Selection of in silico fragmentation software, including commercially or freely available algorithms. Additional algorithms are referenced in the text.

Tools	Fragmentation Method	Compound DB	Type of Interfacce
MS-FINDER	Rule-based (hydrogen rearrangement rules)	15 integrated target DBs plus MINE and PubChem	Windows GUI
CFM-ID	Hybrid rule-based machine learning	KEGG, HMDB	Web application and command line tool
MetFrag	Hybrid rule-based combinatorial	HMDB, KEGG, PubChem	Web application, command line tool,
Mass Frontier	Rule-based (literature reaction mechanisms)	Internal MS database	Windows GUI
ChemDistiller	Fingerprint and spectral machine learning	17 different target databases, 130 Mio compounds total	Command line, web-based output
MAGMa, MAGMa+	Rule-based	PubChem, KEGG, HMDB	Web application, command line tool
CSI:FingerID	Combination of fragmentation trees and machine learning	PubChem and multiple bio databases	Platform independent GUI, command line tool

**Table 6:** Overview of collaborative software and data sharing repositories, major metabolomics repositories and mass spectral sharing initiatives.

Data Sharing	Link	Description
GitHub	github.com	Software development platform
BitBucket	bitbucket.org	Collaborative software sharing
SourceForge	sourceforge.net	Collaborative software sharing
Zenodo	zenodo.org	Open research data repository
Figshare	figshare.com	Online research data repository
Metabolomics Workbench	metabolomicsworkbench.org	Experimental metabolomics data
MetaboLights	ebi.ac.uk/metabolights	European metabolomics repository
OpenMSI	openmsi.nersc.gov	Mass spectral imaging data
MetaSpace	metaspace2020.eu	Mass spectral imaging data
GNPS	gnps.ucsd.edu	Mass spectral data sharing
MassBank	massbank.jp	Mass spectral data sharing
MoNA	massbank.us	Mass spectral sharing community
Norman MassBank	massbank.eu	Mass spectral data sharing
